# Supplementary material for: Action-value processing underlies the role of the dorsal anterior cingulate cortex in performance monitoring during self-regulation of affect
Source: PLoS One. 2022 Aug 30;17(8):e0273376. doi: 10.1371/journal.pone.0273376 (PMC9426889; doi:10.1371/journal.pone.0273376)
Supplement: S1 Methods — (DOCX) [file pone.0273376.s001.docx]

**S1 Methods**

**Image Stimulus Selection**

As reported in prior work[1], 90 implicit induction image stimuli were selected from the International Affective Picture Set (IAPS). Each IAPS image is associated with normative scores (based upon group-level mean measurements from a 9-point Likert scale) of image valence and arousal. We computationally sampled our image subset from the full IAPS image set according to a maximum separation heuristic in which (starting with a randomly selected image) each additional image is selected such that its normative valence and arousal scores exhibit the maximum summed Euclidean distance (in arousal-valence coordinates) to all currently selected images (see S1 Fig). 30 images comprising the cues of the cued-recall/re-experiencing trials were similarly sampled (see S1 Fig). The image sets were then fixed for all participants. Normative valence and arousal scores for all image stimuli, as well as counts and distributions of the affective scores comprising the positive and negative classes used to train the decoding models, are presented in S1–S3 Tables.

**Reliable Stimulus Subset Sampling**

A key question facing affect processing researchers is how to accurately classify stimuli. As valence and arousal are dimensional properties of affect, the natural demarcation of, e.g., positive versus negative, valence is potentially unclear *a priori*. Moreover, the propriety of labeling certain stimuli as neutrally valent is also unclear. Similar questions arise in the labeling of arousal. These questions become particularly relevant when curating stimuli for the training of affect processing decoding models and for comparing performance between decoding models. Training data that cluster in the extremes of an affective dimension may confer a degree of simplicity to the decoding problem that does not necessarily exist in nature. In prior work we described how image stimuli could exhibit reliable properties of positive versus negative affect processing by measuring the degree to which the brain states induced by these stimuli cluster. Stimuli exhibiting canonical two-class induction properties were labeled as part of the ‘Reliable Stimulus Subset’ of the total stimulus set[1].

In this work we modified the original Reliable Stimulus Subset selection algorithm to incorporate a null distribution formed from global permutation testing of the decoding models. For clarity, we summarize the algorithm as follows. For each subject, i, for each stimulus, j, we evaluated the reliability of stimulus j based on the distribution of predictions made for this stimulus by the remaining set of study subjects (n=88). Each stimulus that exhibited prediction accuracy greater than chance according to the binomial distribution (n=88, *α*=0.05) where the null probability was defined as the mean accuracy of all remaining subjects’ permutation tests (SVM fit of beta-series to uniformly randomly assigned class labels averaged over 1000 trials) was identified as ‘reliable’ and added to the subject’s reliable stimulus set, RSS_i_. We then conducted within-subject classification of the RSS using the i^th^ subject’s decoding model and report accuracy on this dataset. Reporting RSS classification accuracy, in addition to classification accuracy on the full dataset, is important for understanding potential performance biases that may exist for curated datasets in which stimuli were hand-selected and, therefore, may possess artificially clustered normative affect scores, the existence of which we have previously reported[1,2].

**References**

1. Bush KA, Gardner J, Privratsky A, Chung M-H, James GA, Kilts CD. Brain States That Encode Perceived Emotion Are Reproducible but Their Classification Accuracy Is Stimulus-Dependent. Frontiers in Human Neuroscience [Internet]. 2018 Jul 2 [cited 2018 Jul 25];12. Available from: https://www.frontiersin.org/article/10.3389/fnhum.2018.00262/full

2. Wilson KA, James GA, Kilts CD, Bush KA. Combining Physiological and Neuroimaging Measures to Predict Affect Processing Induced by Affectively Valent Image Stimuli. Sci Rep. 2020 Dec;10(1):9298.
