# Supplementary figures and images for: Action-value processing underlies the role of the dorsal anterior cingulate cortex in performance monitoring during self-regulation of affect
Source: PLoS One. 2022 Aug 30;17(8):e0273376. doi: 10.1371/journal.pone.0273376 (PMC9426889; doi:10.1371/journal.pone.0273376)

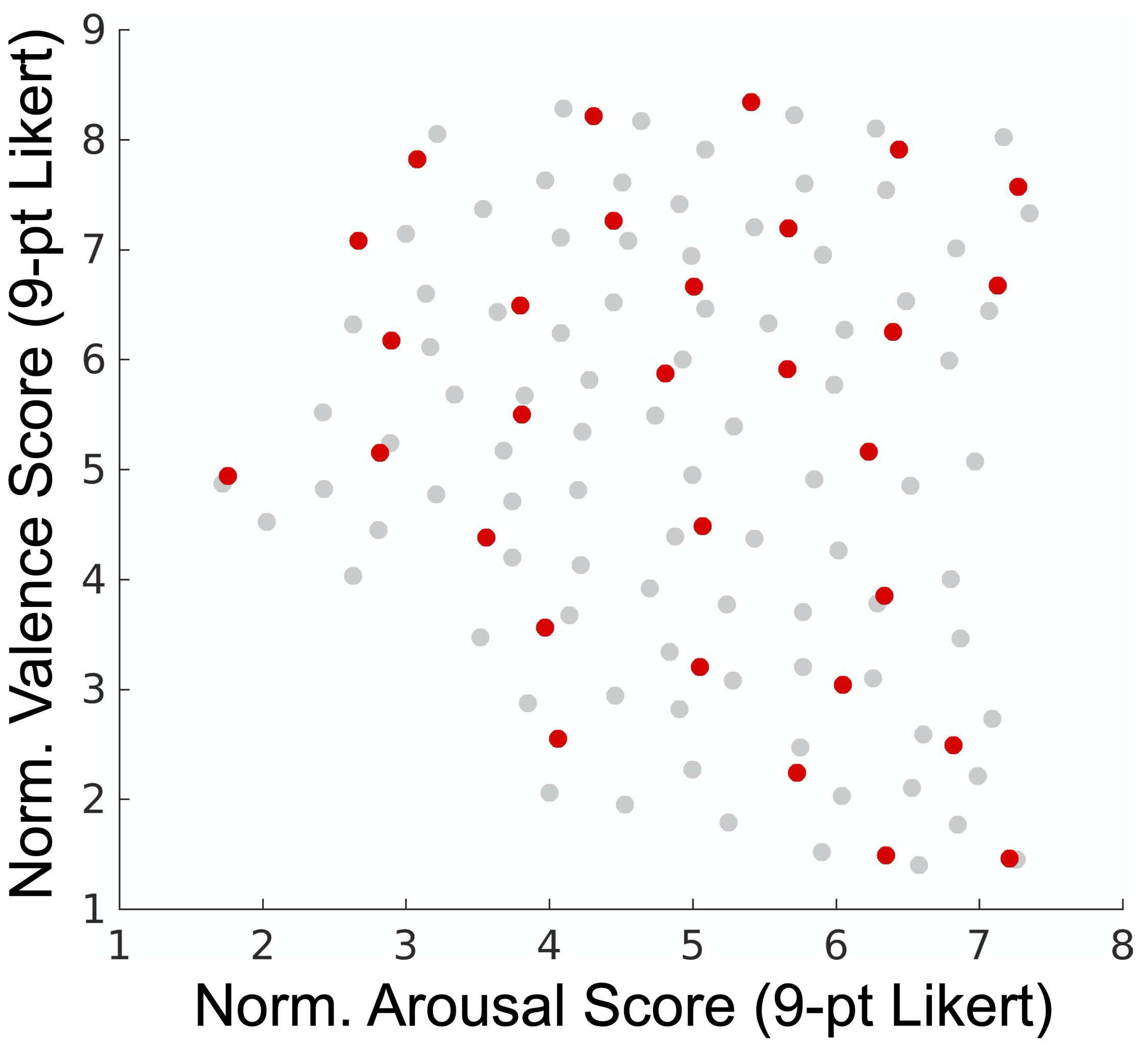

Supplement: S1 Fig — Solid red markers depict (n = 30) individual cue stimuli plotted in coordinates representing mean normative arousal and valence scores. For comparison, solid gray markers depict (n = 90) individual implicit induction stimuli plotted in similar coordinates. (TIF) [file pone.0273376.s002.tif]

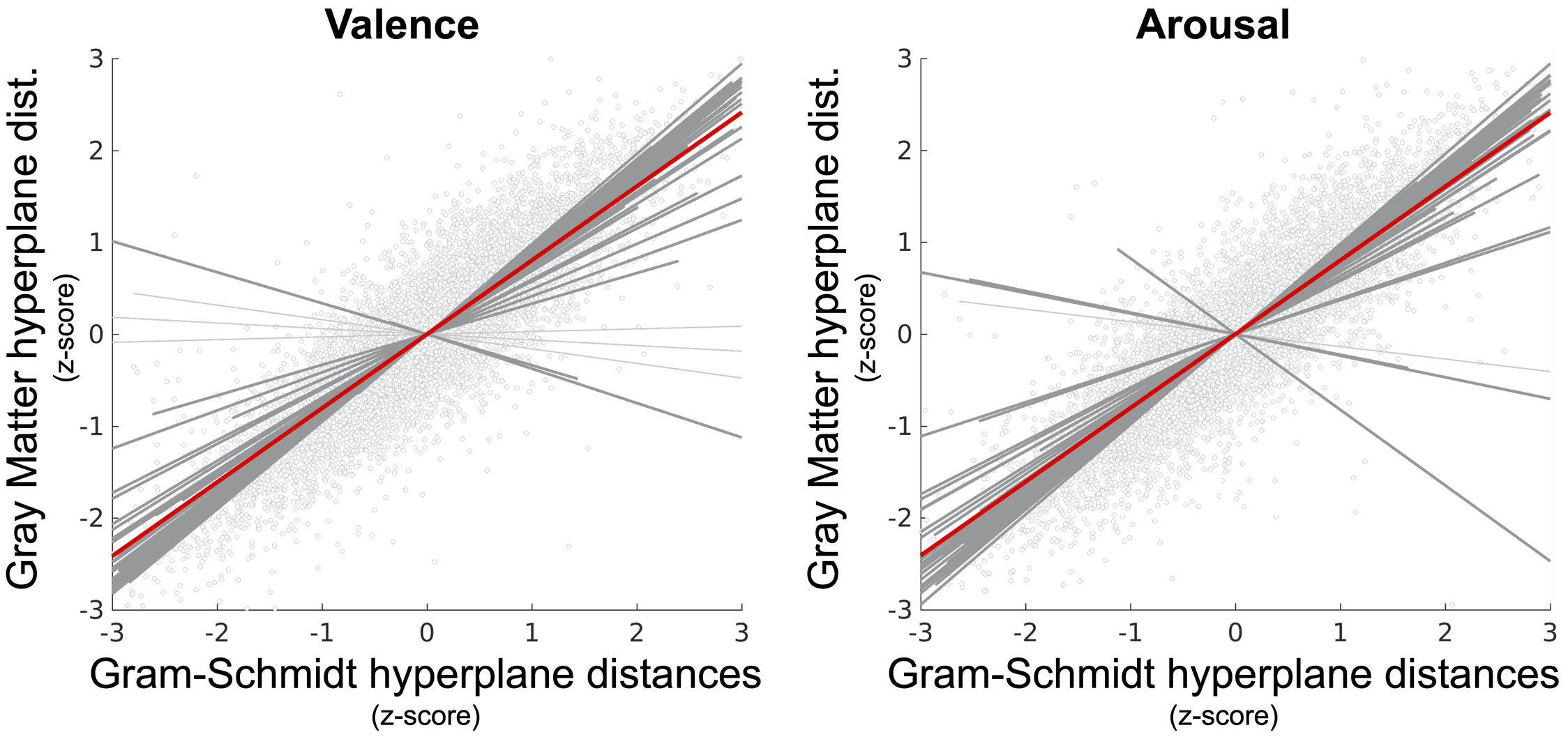

Supplement: S2 Fig — Gram-Schmidt dimensionality reduction projects the original whole-brain gray matter features (n~30,000–40,000) onto an orthogonal basis in which the coordinate dimension is less than or equal to the number of sample features (n≤90). We report the effect size of the reduced dimensional predictions in explaining predictions in the original feature space using a linear mixed-effects model in which random effects are modeled subject-wise. Gray symbols depict individual trials. The bold red line depicts the group-level effect. Bold gray lines depict significant subject-level effects whereas light gray lines depict subject-level effects that were not significant. Valence. The fixed effect (R2 = .71) is significant (p<0.001; t-test; h0: β = 0). Random effects significantly improve effect-size (p<0.05; likelihood ratio test; h0: observed responses generated by fixed-effects only). Arousal. The fixed effect (R2 = .72) is significant (p<0.001; t-test; h0: β = 0). Random effects significantly improve effect-size (p<0.05; likelihood ratio test; h0: observed responses generated by fixed-effects only). (TIF) [file pone.0273376.s003.tif]

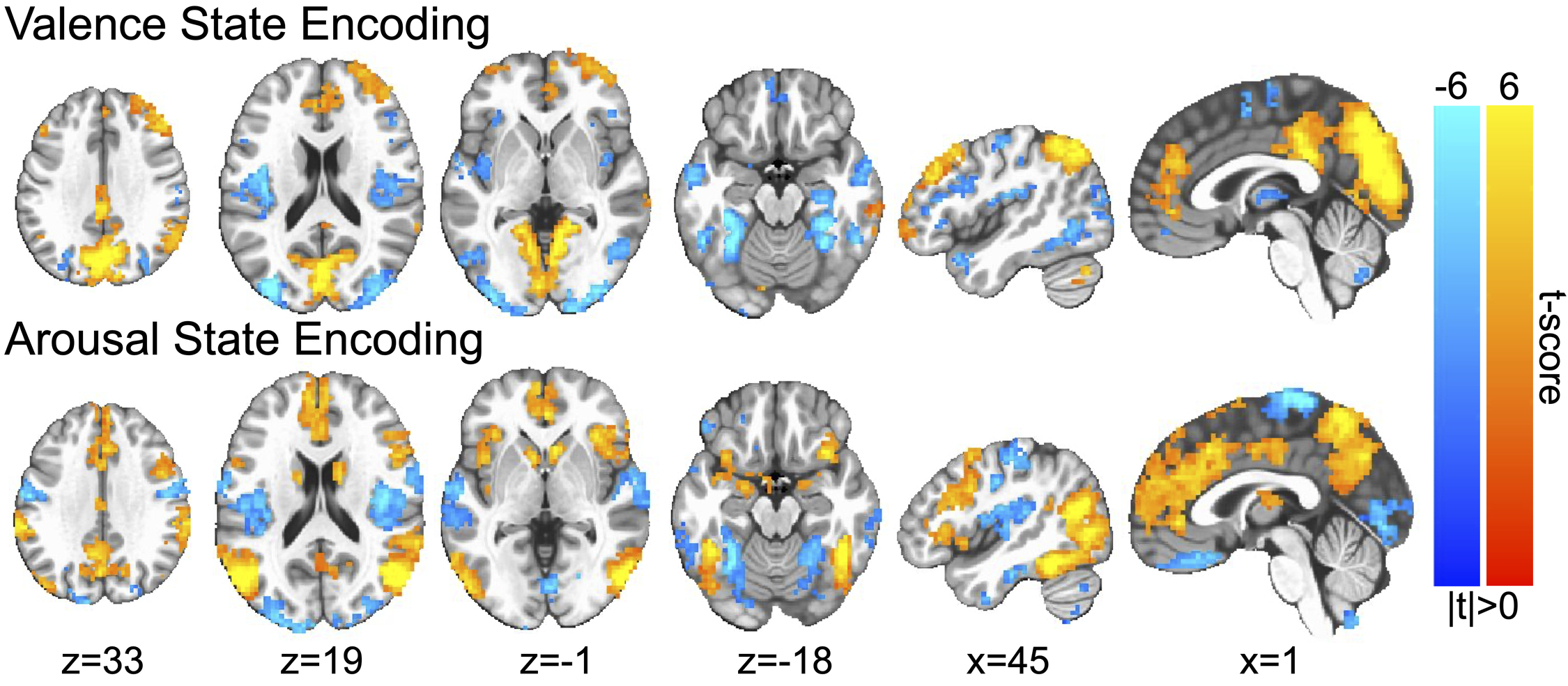

Supplement: S3 Fig — Color gradations indicate the group-level t-scores of the encoding parameters (red indicating positive valence or high arousal, blue indicating negative valence or low arousal). T-scores are presented only for those voxels in which encoding parameters survived global permutation testing (p<0.01, uncorrected, N = 1000 random permutations). Image slices are presented in MNI coordinate space and neurological convention. Maximum voxel intensity is |t| = 6.0, i.e., color saturates for t-scores with absolute values falling above this value. (TIF) [file pone.0273376.s004.tif]

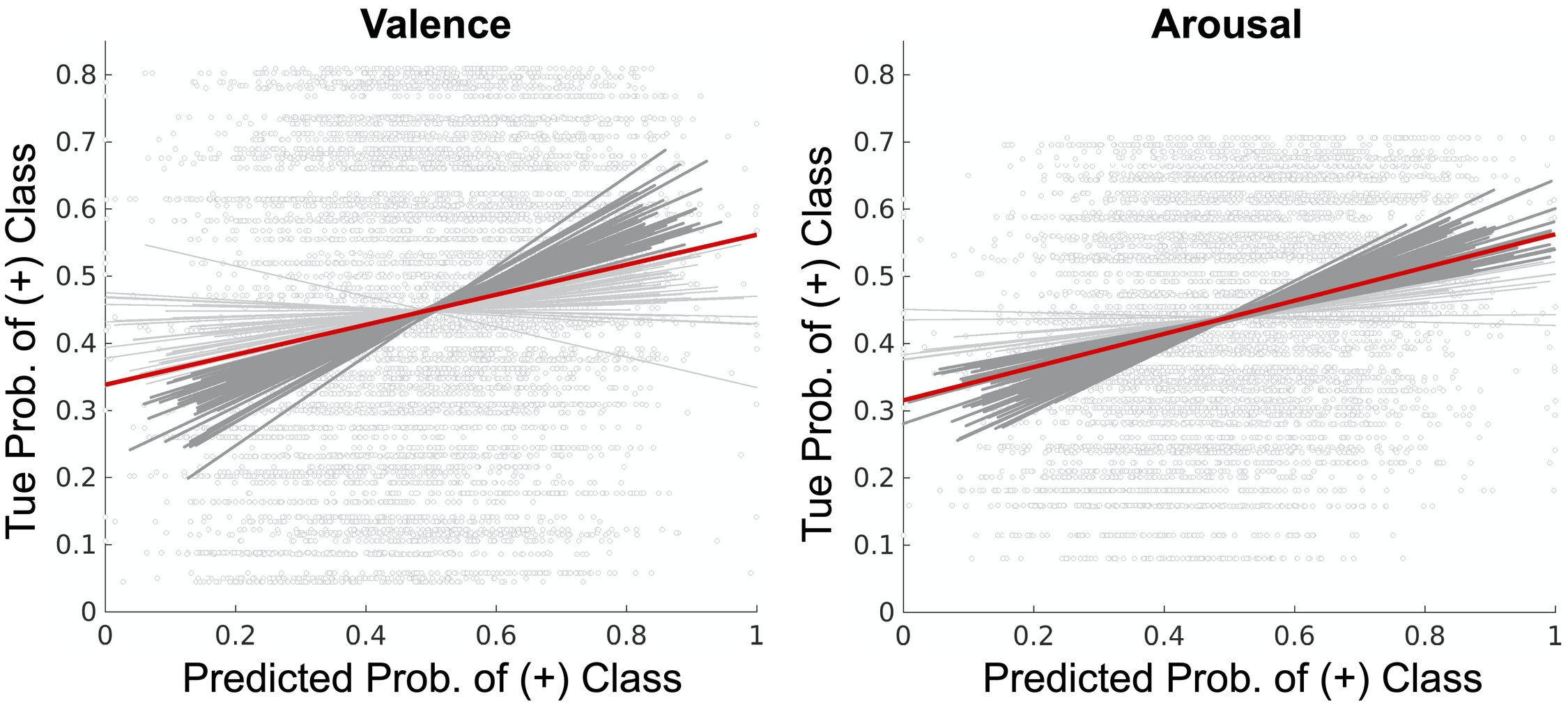

Supplement: S4 Fig — The figure depicts the effect size of Platt-scaled hyperplane distance predictions in explaining the Platt-scaled normative affect scores of IAPS stimuli used to train the support vector machine classifiers, separately for the orthogonal affective dimensions of valence and arousal. Hyperplane distance predictions resulted from within-subject leave-one-out cross-validation. The figure depicts the group-level effects computed using a linear mixed-effects model which modeled random effects subject-wise. Gray symbols depict individual trials. The bold red line depicts the group-level effect. Bold gray lines depict significant subject-level effects whereas light gray lines depict subject-level effects that were not significant. Valence. The fixed effect (R2 = .03) is significant (p<0.001; t-test; h0: β = 0). Random effects significantly improve effect-size (p<0.05; likelihood ratio test; h0: observed responses generated by fixed-effects only). Arousal. The fixed effect (R2 = .07) is significant (p<0.001, t-test; h0: β = 0). Random effects do not significantly improve effect-size. (TIF) [file pone.0273376.s005.tif]

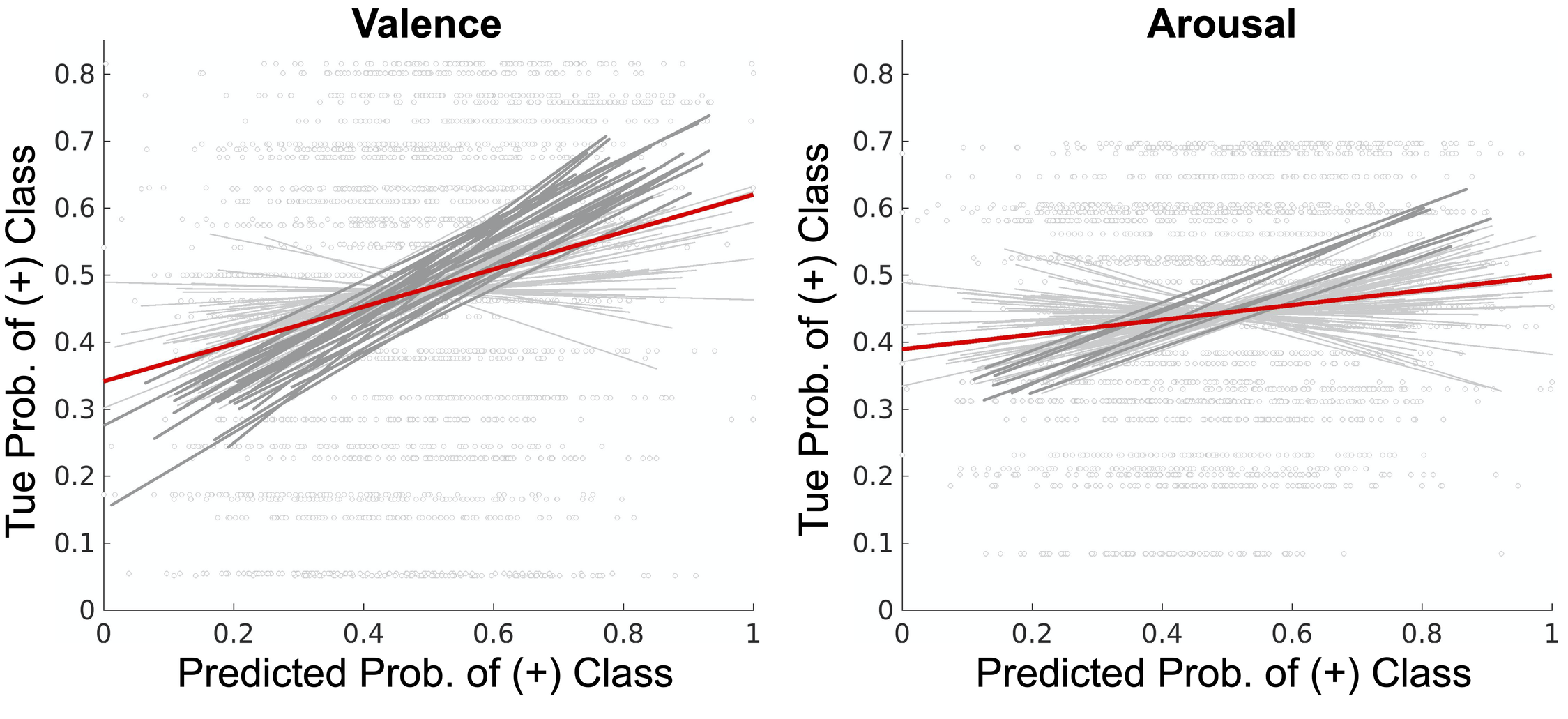

Supplement: S5 Fig — The figure depicts the effect size of Platt-scaled hyperplane distances predicted by the fitted SVMs in explaining the Platt-scaled normative affect scores of IAPS stimuli used as cue stimuli in the cued-recall/re-experiencing affect regulation task. Effect-sizes are reported separately for the orthogonal affective dimensions of valence and arousal. The figure depicts the group-level effects computed using a linear mixed-effects model which modeled random effects subject-wise. Gray symbols depict individual trials. The bold red line depicts the group-level effect. Bold gray lines depict significant subject-level effects whereas light gray lines depict subject-level effects that were not significant. Valence. The fixed effect (R2 = .05) is significant (p<0.001; t-test; h0: β = 0). Random effects do not significantly improve effect size (likelihood ratio test; h0: observed responses generated by fixed-effects only). Arousal. The fixed effect (R2 = .01) is significant (p<0.001; t-test; h0: β = 0). Random effects do not significantly improve effect-size (likelihood ratio test; h0: observed responses generated by fixed-effects only). (TIF) [file pone.0273376.s006.tif]

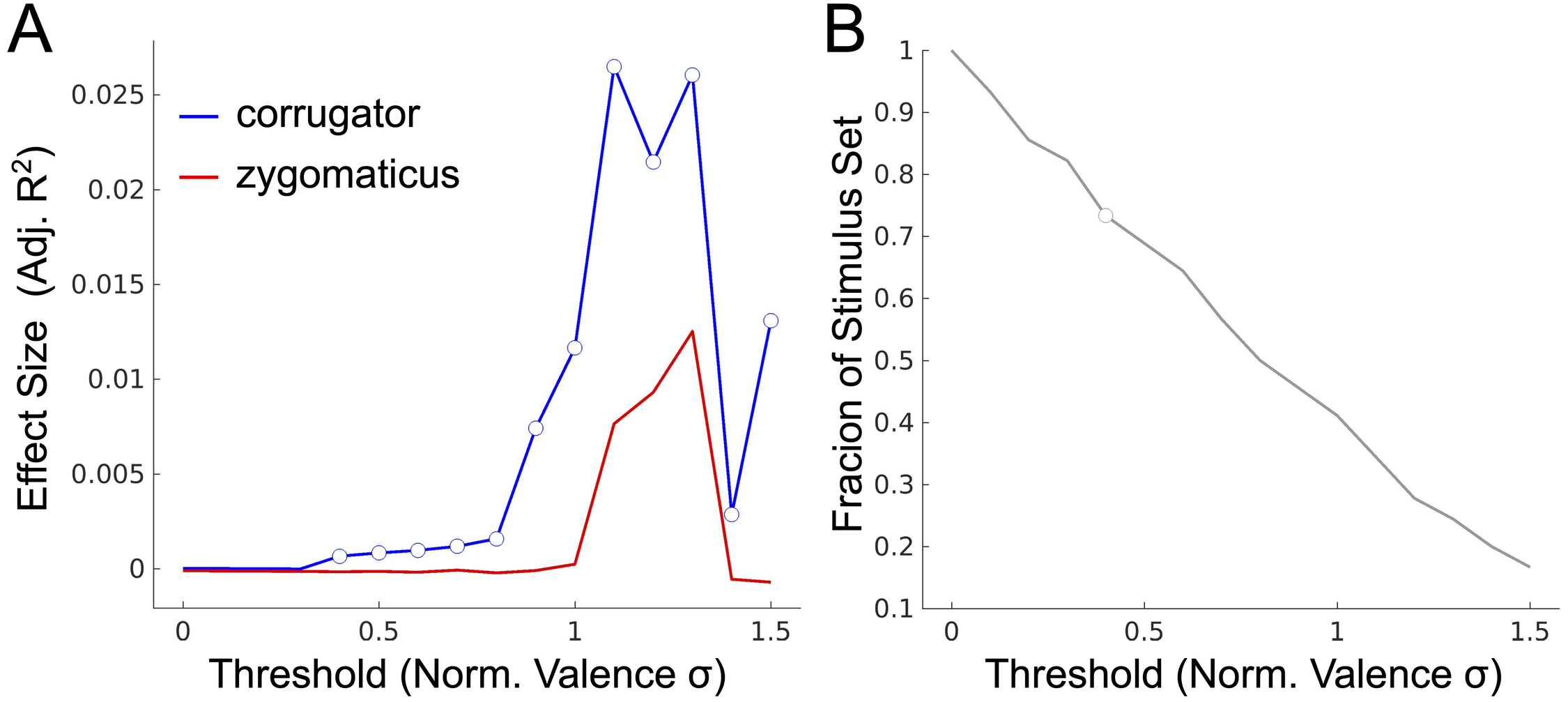

Supplement: S6 Fig — (A) Valence prediction effect-size, measured as adjusted R2, as a function of the polar extremes of affectively valent stimuli used to construct the prediction, plotted separately for facial EMG signals recorded from the corrugator supercilii (blue) and zygomaticus major (red). Polar-extremity is reported as a factor of the standard deviation of the normative valence scores used to threshold stimuli for exclusion from the prediction. The symbols represent thresholds for which the plotted effect-size is statistically significant. (B) The fraction of the total number of image stimuli remaining in the set after thresholding. The symbol denotes the minimum threshold level for which the corrugator signal significantly predicted normative valence score of the remaining stimuli. (TIF) [file pone.0273376.s007.tif]

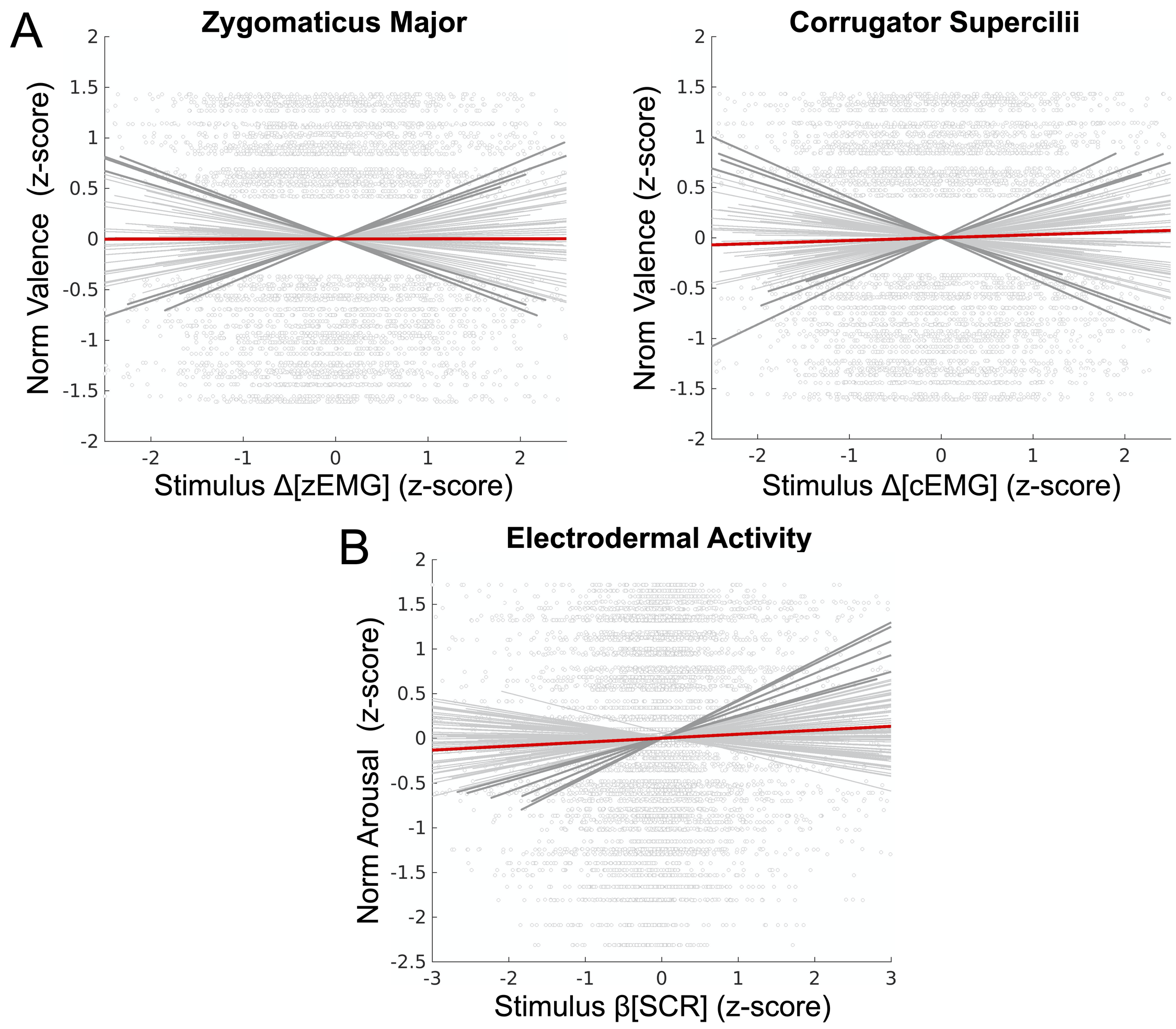

Supplement: S7 Fig — (A) Facial electromyography based prediction of normative valence scores of the stimulus set (thresholded .4σ, see S6 Fig). The group-level fixed effect (R2 = .0002) of zygomaticus major, zEMG, differences between pre- and post-stimulus rectified signals is not significant (p = .78; t-test; h0: β = 0). The group-level fixed effect (R2 = .0007) of corrugator supercilii, cEMG, is significant (p = .024; t-test; h0: β = 0). Random effects did not significantly improve effect-size (p<0.05; likelihood ratio test; h0: observed responses generated by fixed-effects only). (B) Electrodermal activity based prediction of normative arousal scores of the full (i.e., unthresholded) stimulus set. The group-level fixed effect (R2 = .002) of the skin conductance response, SCR, beta-series is significant (p<0.0001; t-test; h0: β = 0). Random effects did not significantly improve effect-size (p>0.05; likelihood ratio test; h0: observed responses generated by fixed-effects only). In both panels, gray symbols represent individual trials, bold gray lines depict significant subject-level effects, and light gray lines depict subject-level effects that were not significant. (TIF) [file pone.0273376.s008.tif]

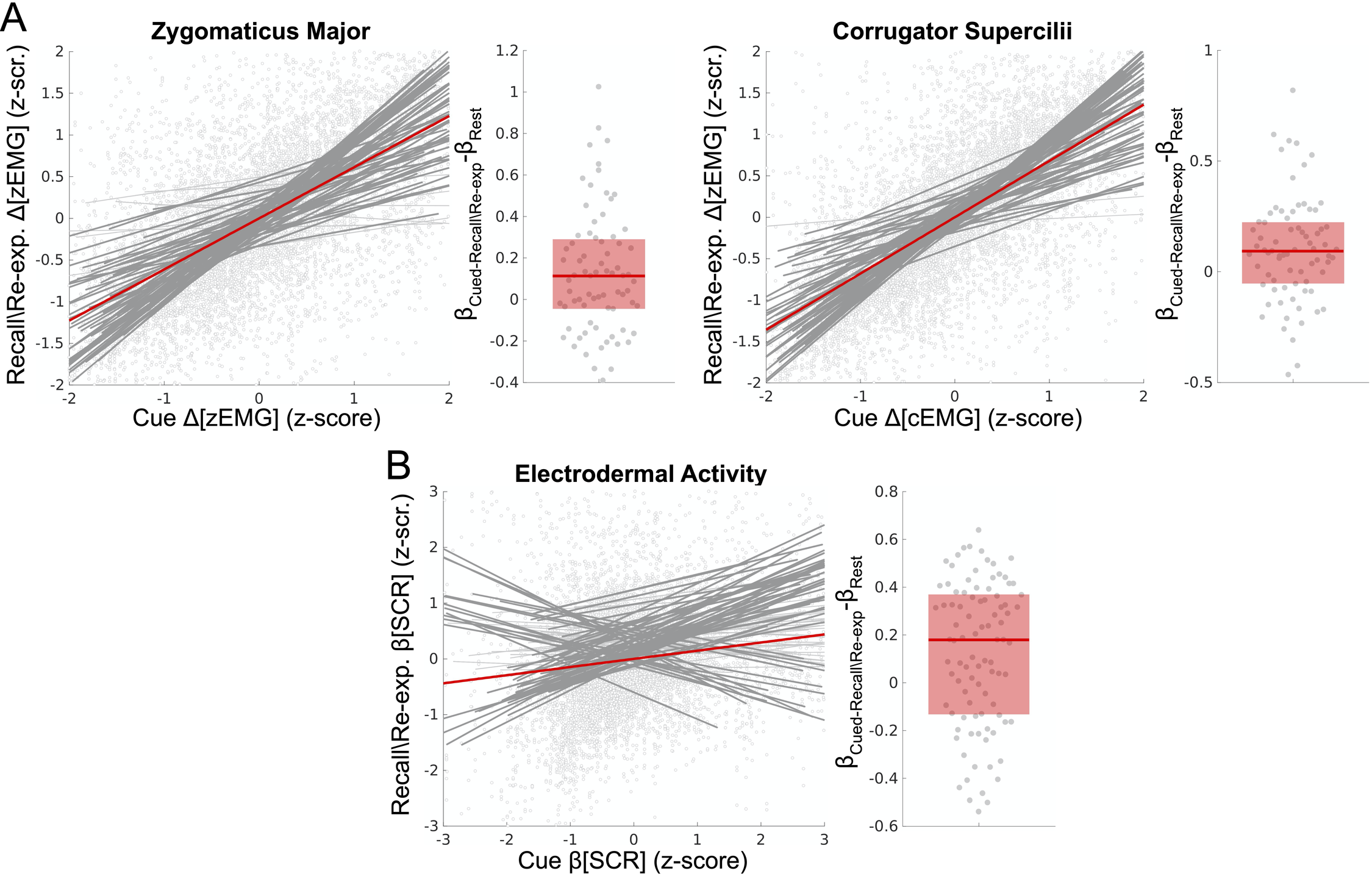

Supplement: S8 Fig — The figure depicts the effect sizes of cued affect processing in explaining affect processing occurring during recall/re-experiencing (controlling for the duration of the 4 repeated measurements of recall/re-experience per each measurement of cue) for each of three unique psychophysiological measurements: facial electromyography of the zygomaticus major (zEMG), facial electromyography of the corrugator supercilii (cEMG), and electrodermal activity measured as galvanic skin conductance response (SCR). Here affect processing induction measurements are standardized measurements specific to each measurement modality (differences between pre- and post-stimulus for electromyography or modeled betas for skin conductance response). Scatterplots depict the group-level effects computed using linear mixed-effects models which model random effects subject-wise. Bold red lines depict group-level fixed-effects of the cue affect. Bold gray lines depict significant subject-level effects whereas light gray lines depict subject-level effects that were not significant. The figure’s boxplots depict group-level affect processing induction measured during the cued-recall/re-experiencing task in comparison to affect processing induction constructed from the resting state task. The bold red line depicts the group median difference in effect size between cued-recall/re-experiencing and resting state. The red box depicts the 25-75th percentiles of effect size difference. Note, we measured zEMG and cEMG for CTM subjects (n = 56) only. We measured SCR for all subjects. (A) The fixed effect (R2 = .45) of zEMG is significant (p<0.001; t-test; h0: β = 0). Random effects significantly improve effect-size (p<0.05; likelihood ratio test; h0: observed responses generated by fixed-effects only). Cued-recall/re-experiencing affect processing induction effects are significantly greater than that of resting state control condition effects (p<0.002; Wilcoxon signed rank; h0: βIN- βRST = 0). The control du [file pone.0273376.s009.tif]

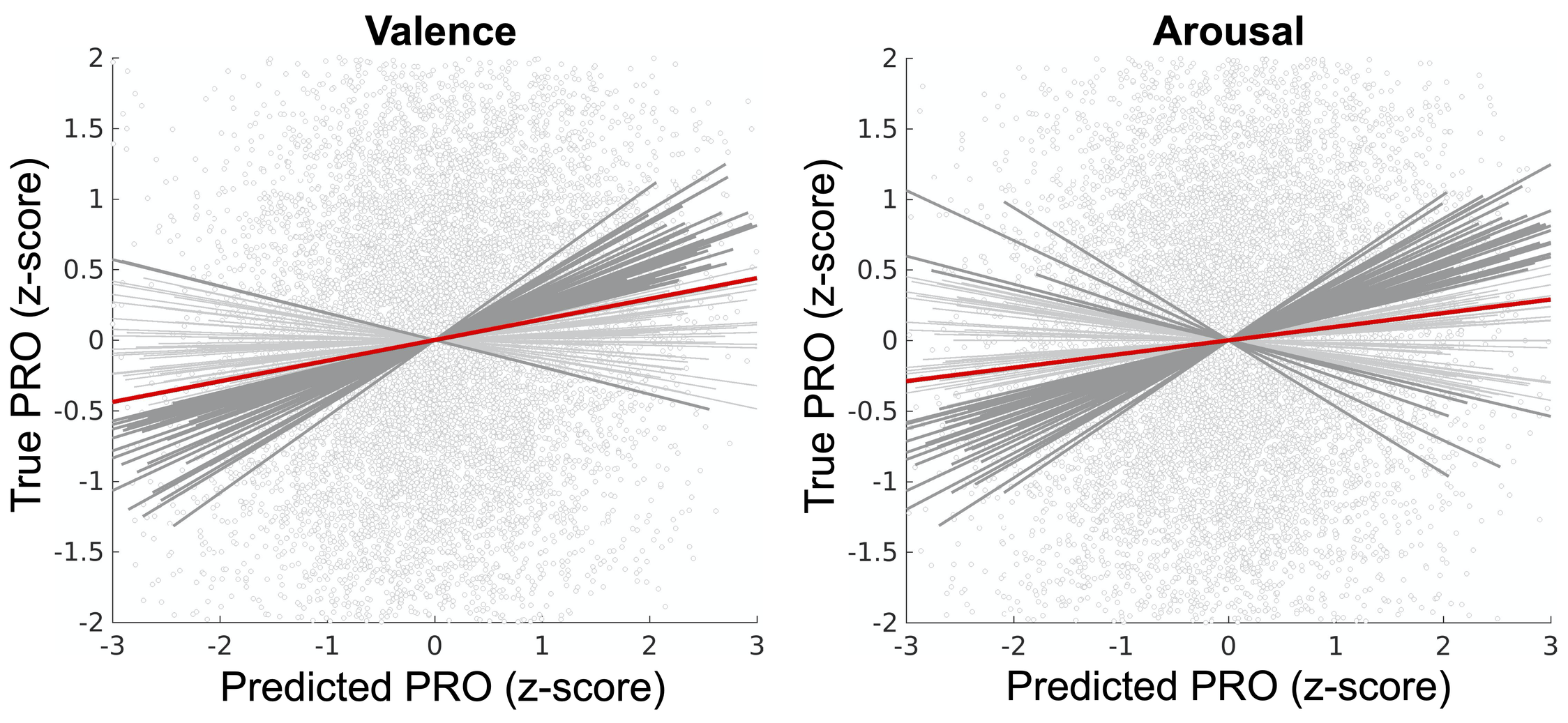

Supplement: S9 Fig — Scatterplots depict the group-level effects computed using linear mixed-effects models which model random effects subject-wise. Bold red lines depict group-level fixed-effects of the models’ predictions of the true PRO. Bold gray lines depict significant subject-level effects whereas light gray lines depict subject-level effects that were not significant. Valence. The fixed effect (R2 = .039) is significant (p<0.001; t-test; h0: β = 0). Random effects significantly improve effect-size (p<0.05; likelihood ratio test; h0: observed responses generated by fixed-effects only). Arousal. The effect (R2 = .031) is significant (p<0.001; t-test; h0: β = 0). Random effects significantly improve effect-size (p<0.05; likelihood ratio test; h0: observed responses generated by fixed-effects only). (TIF) [file pone.0273376.s010.tif]

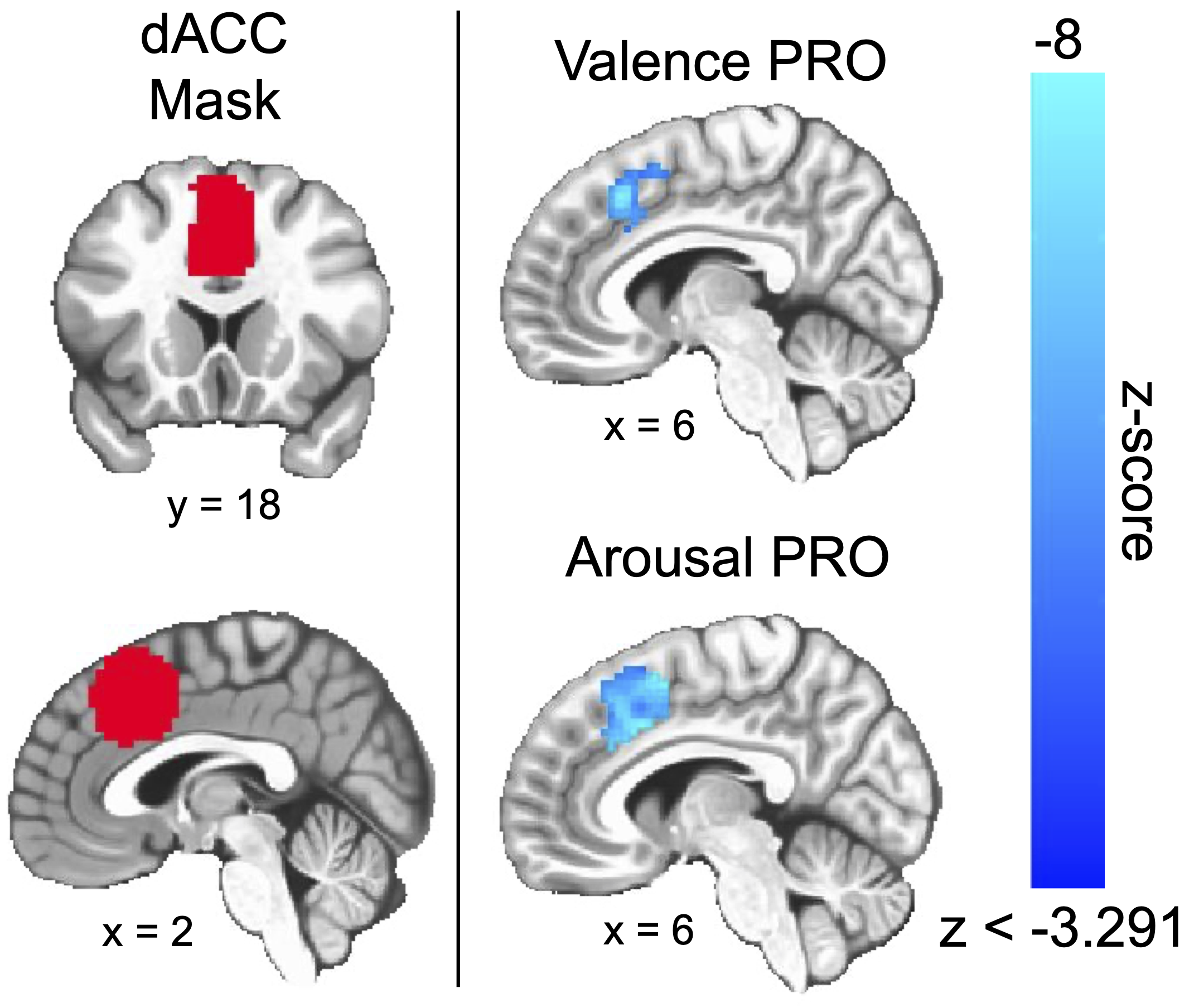

Supplement: S10 Fig — The figure depicts slices in MNI coordinate space and neurological convention (image left equals participant left) that highlight the strongest effects of PRO (compare to Fig 3). The figure depicts voxel intensities as colors -8 < z< -3.291. Color saturates for z-scores below minimum intensity and no color is presented for z-scores above -3.291. The figure depicts only valence derived clusters having ≥ 15.3 contiguous voxels (measured as face wise nearest neighbors, i.e., NN = 1) or arousal derived clusters having ≥ 15.8 contiguous voxels. (TIF) [file pone.0273376.s011.tif]
