# Supplementary material for: Action-value processing underlies the role of the dorsal anterior cingulate cortex in performance monitoring during self-regulation of affect
Source: PLoS One. 2022 Aug 30;17(8):e0273376. doi: 10.1371/journal.pone.0273376 (PMC9426889; doi:10.1371/journal.pone.0273376)
Supplement: S1 Table — (DOCX) [file pone.0273376.s012.docx]

**S1 Table. Image stimuli IAPS identifiers and normative valence and arousal scores separated by trial type.**

| **#** | **Trial Type** | **IAPS ID** | **Valence** | **Arousal** |
| --- | --- | --- | --- | --- |
| **1** | Implicit Induction | 1022 | 4.26 | 6.02 |
| **2** | Implicit Induction | 1050 | 3.46 | 6.87 |
| **3** | Implicit Induction | 1301 | 3.7 | 5.77 |
| **4** | Implicit Induction | 1333 | 6.11 | 3.17 |
| **5** | Implicit Induction | 1620 | 7.37 | 3.54 |
| **6** | Implicit Induction | 1750 | 8.28 | 4.1 |
| **7** | Implicit Induction | 1810 | 6.52 | 4.45 |
| **8** | Implicit Induction | 1931 | 4 | 6.8 |
| **9** | Implicit Induction | 2020 | 5.68 | 3.34 |
| **10** | Implicit Induction | 2040 | 8.17 | 4.64 |
| **11** | Implicit Induction | 2058 | 7.91 | 5.09 |
| **12** | Implicit Induction | 2095 | 1.79 | 5.25 |
| **13** | Implicit Induction | 2205 | 1.95 | 4.53 |
| **14** | Implicit Induction | 2217 | 6.24 | 4.08 |
| **15** | Implicit Induction | 2222 | 7.11 | 4.08 |
| **16** | Implicit Induction | 2271 | 4.2 | 3.74 |
| **17** | Implicit Induction | 2279 | 4.71 | 3.74 |
| **18** | Implicit Induction | 2302 | 6.43 | 3.64 |
| **19** | Implicit Induction | 2351 | 5.49 | 4.74 |
| **20** | Implicit Induction | 2352 | 6.94 | 4.99 |
| **21** | Implicit Induction | 2520 | 4.13 | 4.22 |
| **22** | Implicit Induction | 2540 | 7.63 | 3.97 |
| **23** | Implicit Induction | 2722 | 3.47 | 3.52 |
| **24** | Implicit Induction | 2795 | 3.92 | 4.7 |
| **25** | Implicit Induction | 3000 | 1.45 | 7.26 |
| **26** | Implicit Induction | 3015 | 1.52 | 5.9 |
| **27** | Implicit Induction | 3102 | 1.4 | 6.58 |
| **28** | Implicit Induction | 3250 | 3.78 | 6.29 |
| **29** | Implicit Induction | 3310 | 4.37 | 5.43 |
| **30** | Implicit Induction | 3500 | 2.21 | 6.99 |
| **31** | Implicit Induction | 4220 | 6.6 | 5.18 |
| **32** | Implicit Induction | 4235 | 5.39 | 5.29 |
| **33** | Implicit Induction | 4490 | 6.27 | 6.06 |
| **34** | Implicit Induction | 4503 | 6 | 4.93 |
| **35** | Implicit Induction | 4531 | 5.81 | 4.28 |
| **36** | Implicit Induction | 4550 | 4.95 | 5 |
| **37** | Implicit Induction | 4597 | 6.95 | 5.91 |
| **38** | Implicit Induction | 4598 | 6.33 | 5.53 |
| **39** | Implicit Induction | 4619 | 6.46 | 5.09 |
| **40** | Implicit Induction | 4626 | 7.6 | 5.78 |
| **41** | Implicit Induction | 4641 | 7.2 | 5.43 |
| **42** | Implicit Induction | 4649 | 5.77 | 5.99 |
| **43** | Implicit Induction | 4770 | 4.91 | 5.85 |
| **44** | Implicit Induction | 4800 | 6.44 | 7.07 |
| **45** | Implicit Induction | 5010 | 7.14 | 3 |
| **46** | Implicit Induction | 5020 | 6.32 | 2.63 |
| **47** | Implicit Induction | 5395 | 5.34 | 4.23 |
| **48** | Implicit Induction | 5750 | 6.6 | 3.14 |
| **49** | Implicit Induction | 5760 | 8.05 | 3.22 |
| **50** | Implicit Induction | 5833 | 8.22 | 5.71 |
| **51** | Implicit Induction | 5950 | 5.99 | 6.79 |
| **52** | Implicit Induction | 5982 | 7.61 | 4.51 |
| **53** | Implicit Induction | 6300 | 2.59 | 6.61 |
| **54** | Implicit Induction | 6550 | 2.73 | 7.09 |
| **55** | Implicit Induction | 6563 | 1.77 | 6.85 |
| **56** | Implicit Induction | 6930 | 4.39 | 4.88 |
| **57** | Implicit Induction | 7031 | 4.52 | 2.03 |
| **58** | Implicit Induction | 7043 | 5.17 | 3.68 |
| **59** | Implicit Induction | 7100 | 5.24 | 2.89 |
| **60** | Implicit Induction | 7175 | 4.87 | 1.72 |
| **61** | Implicit Induction | 7211 | 4.81 | 4.2 |
| **62** | Implicit Induction | 7217 | 4.82 | 2.43 |
| **63** | Implicit Induction | 7224 | 4.45 | 2.81 |
| **64** | Implicit Induction | 7285 | 5.67 | 3.83 |
| **65** | Implicit Induction | 7480 | 7.08 | 4.55 |
| **66** | Implicit Induction | 7490 | 5.52 | 2.42 |
| **67** | Implicit Induction | 7492 | 7.41 | 4.91 |
| **68** | Implicit Induction | 8030 | 7.33 | 7.35 |
| **69** | Implicit Induction | 8158 | 6.53 | 6.49 |
| **70** | Implicit Induction | 8160 | 5.07 | 6.97 |
| **71** | Implicit Induction | 8186 | 7.01 | 6.84 |
| **72** | Implicit Induction | 8190 | 8.1 | 6.28 |
| **73** | Implicit Induction | 8200 | 7.54 | 6.35 |
| **74** | Implicit Induction | 8231 | 3.77 | 5.24 |
| **75** | Implicit Induction | 8475 | 4.85 | 6.52 |
| **76** | Implicit Induction | 9102 | 3.34 | 4.84 |
| **77** | Implicit Induction | 9120 | 3.2 | 5.77 |
| **78** | Implicit Induction | 9163 | 2.1 | 6.53 |
| **79** | Implicit Induction | 9184 | 2.47 | 5.75 |
| **80** | Implicit Induction | 9220 | 2.06 | 4 |
| **81** | Implicit Induction | 9254 | 2.03 | 6.04 |
| **82** | Implicit Induction | 9331 | 2.87 | 3.85 |
| **83** | Implicit Induction | 9360 | 4.03 | 2.63 |
| **84** | Implicit Induction | 9390 | 3.67 | 4.14 |
| **85** | Implicit Induction | 9415 | 2.82 | 4.91 |
| **86** | Implicit Induction | 9426 | 3.08 | 5.28 |
| **87** | Implicit Induction | 9435 | 2.27 | 5 |
| **88** | Implicit Induction | 9622 | 3.1 | 6.26 |
| **89** | Implicit Induction | 9700 | 4.77 | 3.21 |
| **90** | Implicit Induction | 9832 | 2.94 | 4.46 |
| **1** | Cued-Recall/Re-exp. | 1460 | 8.21 | 4.31 |
| **2** | Cued-Recall/Re-exp. | 1610 | 7.82 | 3.08 |
| **3** | Cued-Recall/Re-exp. | 1630 | 7.26 | 4.45 |
| **4** | Cued-Recall/Re-exp. | 1710 | 8.34 | 5.41 |
| **5** | Cued-Recall/Re-exp. | 2060 | 6.49 | 3.8 |
| **6** | Cued-Recall/Re-exp. | 2210 | 4.38 | 3.56 |
| **7** | Cued-Recall/Re-exp. | 2320 | 6.17 | 2.9 |
| **8** | Cued-Recall/Re-exp. | 3063 | 1.49 | 6.35 |
| **9** | Cued-Recall/Re-exp. | 3170 | 1.46 | 7.21 |
| **10** | Cued-Recall/Re-exp. | 4008 | 5.91 | 5.66 |
| **11** | Cued-Recall/Re-exp. | 4470 | 5.87 | 4.81 |
| **12** | Cued-Recall/Re-exp. | 4668 | 6.67 | 7.13 |
| **13** | Cued-Recall/Re-exp. | 5000 | 7.08 | 2.67 |
| **14** | Cued-Recall/Re-exp. | 5510 | 5.15 | 2.82 |
| **15** | Cued-Recall/Re-exp. | 5623 | 7.19 | 5.67 |
| **16** | Cued-Recall/Re-exp. | 5920 | 5.16 | 6.23 |
| **17** | Cued-Recall/Re-exp. | 5972 | 3.85 | 6.34 |
| **18** | Cued-Recall/Re-exp. | 6231 | 2.49 | 6.82 |
| **19** | Cued-Recall/Re-exp. | 7010 | 4.94 | 1.76 |
| **20** | Cued-Recall/Re-exp. | 7283 | 5.5 | 3.81 |
| **21** | Cued-Recall/Re-exp. | 8033 | 6.66 | 5.01 |
| **22** | Cued-Recall/Re-exp. | 8185 | 7.57 | 7.27 |
| **23** | Cued-Recall/Re-exp. | 8341 | 6.25 | 6.4 |
| **24** | Cued-Recall/Re-exp. | 8501 | 7.91 | 6.44 |
| **25** | Cued-Recall/Re-exp. | 9000 | 2.55 | 4.06 |
| **26** | Cued-Recall/Re-exp. | 9090 | 3.56 | 3.97 |
| **27** | Cued-Recall/Re-exp. | 9145 | 3.2 | 5.05 |
| **28** | Cued-Recall/Re-exp. | 9322 | 2.24 | 5.73 |
| **29** | Cued-Recall/Re-exp. | 9402 | 4.48 | 5.07 |
| **30** | Cued-Recall/Re-exp. | 9623 | 3.04 | 6.05 |
