# Supplementary material for: Action-value processing underlies the role of the dorsal anterior cingulate cortex in performance monitoring during self-regulation of affect
Source: PLoS One. 2022 Aug 30;17(8):e0273376. doi: 10.1371/journal.pone.0273376 (PMC9426889; doi:10.1371/journal.pone.0273376)
Supplement: S2 Table — (DOCX) [file pone.0273376.s013.docx]

**S2 Table. Implicit induction stimuli class counts.**

|  | **Valence**  **n=** | **Arousal**  **n=** |
| --- | --- | --- |
| **Class** |  |  |
| **+** | 45 | 44 |
| **-** | 45 | 46 |
