# Supplementary material for: Action-value processing underlies the role of the dorsal anterior cingulate cortex in performance monitoring during self-regulation of affect
Source: PLoS One. 2022 Aug 30;17(8):e0273376. doi: 10.1371/journal.pone.0273376 (PMC9426889; doi:10.1371/journal.pone.0273376)
Supplement: S3 Table — (DOCX) [file pone.0273376.s014.docx]

**S3 Table. Implicit induction stimuli class normative affect score distributions.**

|  | **Valence**  **mean (s.d.)** | **Arousal**  **mean (s.d.)** |
| --- | --- | --- |
| **Class** |  |  |
| **+** | 6.66 (.91) | 6.09 (.68) |
| **-** | 3.39 (1.10) | 3.82 (.83) |
