# Supplementary material for: Action-value processing underlies the role of the dorsal anterior cingulate cortex in performance monitoring during self-regulation of affect
Source: PLoS One. 2022 Aug 30;17(8):e0273376. doi: 10.1371/journal.pone.0273376 (PMC9426889; doi:10.1371/journal.pone.0273376)
Supplement: S4 Table — (DOCX) [file pone.0273376.s015.docx]

**S4 Table. Support vector machine prediction performance.**

|  | **Valence**  **Grp Avg. Acc. (95% CI)** | **Arousal**  **Grp Avg. Acc. (95% CI)** |
| --- | --- | --- |
| **Set of IAPS Stimuli Predicted** |  |  |
| **Full Stim. Set (FSS)** | .56 (.54,.57) | .59 (.58,.61) |
| **Reliable Stim. Subset (RSS)** | .77 (.75,.79) | .76 (.74,.77) |
