# Supplementary material for: Action-value processing underlies the role of the dorsal anterior cingulate cortex in performance monitoring during self-regulation of affect
Source: PLoS One. 2022 Aug 30;17(8):e0273376. doi: 10.1371/journal.pone.0273376 (PMC9426889; doi:10.1371/journal.pone.0273376)
Supplement: S5 Table — Q Performance depicts the median action-value advantage of on-policy control versus a random policy. Policy Error depicts median squared error between the on-policy action and the optimal action. Gray cells depict the cells selected as the parameters for this experiment (see Main Manuscript Methods: Control Performance Evaluation Monitoring). Note, all parameter combinations in the Q Performance represent significant action-value advantages for on-policy control (p<0.05; Wilcoxon rank-sum test; h0: μ1-μ2 = 0). Valence. Selected parameters: discount factor, γ = 0.9; fraction of action, fa = 0.2. Arousal. Selected parameters: discount factor, γ = 1.0; fraction of action, fa = 0.2. (DOCX) [file pone.0273376.s016.docx]

**S5 Table. Validation of inter-subject ensemble moment-to-moment estimates of expected value of control (EVC) within the dACC based upon neural activations falling outside the medial frontal cortex (mFC) and selection of optimal EVC parameters.**

| **Valence** | | | | | | | | |  | **Arousal** | | | | | | | | |
| --- | --- | --- | --- | --- | --- | --- | --- | --- | --- | --- | --- | --- | --- | --- | --- | --- | --- | --- |
| **Q Performance** | | | |  | **Policy Error** | | | |  | **Q Performance** | | | |  | **Policy Error** | | | |
|  | *Fraction of Action* | | |  |  | *Fraction of Action* | | |  |  | *Fraction of Action* | | |  |  | *Fraction of Action* | | |
| *γ* | 0 | 0.2 | 0.4 |  | *γ* | 0 | 0.2 | 0.4 |  | *γ* | 0 | 0.2 | 0.4 |  | *γ* | 0 | 0.2 | 0.4 |
| 0 | .0389 | .1438 | .2303 |  | 0 | .3516 | .3293 | 0.3293 |  | 0 | .0436 | .1457 | .2350 |  | 0 | .3409 | .3395 | .3395 |
| 0.1 | .0374 | .1380 | .2178 |  | 0.1 | .3451 | .3293 | 0.3293 |  | 0.1 | .0420 | .1440 | .2275 |  | 0.1 | .3401 | .3395 | .3395 |
| 0.2 | .0376 | .1333 | .2107 |  | 0.2 | .3482 | .3293 | 0.3293 |  | 0.2 | .0396 | .1375 | .2153 |  | 0.2 | .3407 | .3395 | .3395 |
| 0.3 | .0359 | .1215 | .1975 |  | 0.3 | .3463 | .3293 | 0.3293 |  | 0.3 | .0400 | .1310 | .2039 |  | 0.3 | .3408 | .3395 | .3395 |
| 0.4 | .0344 | .1154 | .1845 |  | 0.4 | .3405 | .3293 | 0.3293 |  | 0.4 | .0400 | .1236 | .1937 |  | 0.4 | .3409 | .3395 | .3395 |
| 0.5 | .0283 | .1116 | .1720 |  | 0.5 | .3428 | .3293 | 0.3293 |  | 0.5 | .0379 | .1151 | .1801 |  | 0.5 | .3425 | .3395 | .3395 |
| 0.6 | .0313 | .0994 | .1600 |  | 0.6 | .3488 | .3293 | 0.3293 |  | 0.6 | .0380 | .1090 | .1699 |  | 0.6 | .3419 | .3395 | .3395 |
| 0.7 | .0270 | .0928 | .1459 |  | 0.7 | .3674 | .3293 | 0.3293 |  | 0.7 | .0380 | .1025 | .1580 |  | 0.7 | .3471 | .3395 | .3395 |
| 0.8 | .0248 | .0842 | .1329 |  | 0.8 | .3852 | .3295 | 0.3293 |  | 0.8 | .0332 | .0957 | .1434 |  | 0.8 | .3463 | .3395 | .3395 |
| 0.9 | .0223 | .0786 | .1190 |  | 0.9 | .4920 | .3293 | 0.3293 |  | 0.9 | .0330 | .0875 | .1343 |  | 0.9 | .3818 | .3395 | .3395 |
| 1.0 | .0221 | .0717 | .1084 |  | 1.0 | .4835 | .3338 | 0.3293 |  | 1.0 | .0320 | .0792 | .1229 |  | 1.0 | .3828 | .3395 | .3395 |

Q Performance depicts the median action-value advantage of on-policy control versus a random policy. Policy Error depicts median squared error between the on-policy action and the optimal action. Gray cells depict the cells selected as the parameters for this experiment (see Main Manuscript Methods: Control Performance Evaluation Monitoring). Note, all parameter combinations in the Q Performance represent significant action-value advantages for on-policy control (p<0.05; Wilcoxon rank-sum test; h_0_: μ_1_-μ_2_=0). **Valence.** Selected parameters: discount factor, γ=0.9; fraction of action, f_a_=0.2. **Arousal.** Selected parameters: discount factor, γ=1.0; fraction of action, f_a_=0.2.
