# Supplementary material for: Action-value processing underlies the role of the dorsal anterior cingulate cortex in performance monitoring during self-regulation of affect
Source: PLoS One. 2022 Aug 30;17(8):e0273376. doi: 10.1371/journal.pone.0273376 (PMC9426889; doi:10.1371/journal.pone.0273376)
Supplement: S6 Table — *p<0.05, **p<0.01, ***p<0.001. (DOCX) [file pone.0273376.s017.docx]

**S6 Table. Summary of bivariate correlation coefficients calculated between each of the primary control performance evaluation models compared in this study**.

| **Valence** | | | |  | **Arousal** | | | |
| --- | --- | --- | --- | --- | --- | --- | --- | --- |
| **Correlations (R)** | | | |  | **Correlations (R)** | | | |
|  | nEVC | PRO | Error |  |  | nEVC | PRO | Error |
| nEVC |  | .0363*** | -.0506*** |  | nEVC |  | .0552*** | -.0864*** |
| PRO |  |  | -.0575*** |  | PRO |  |  | -.0207* |
| Error |  |  |  |  | Error |  |  |  |

*p<0.05, **p<0.01, ***p<0.001
