# Supplementary material for: Action-value processing underlies the role of the dorsal anterior cingulate cortex in performance monitoring during self-regulation of affect
Source: PLoS One. 2022 Aug 30;17(8):e0273376. doi: 10.1371/journal.pone.0273376 (PMC9426889; doi:10.1371/journal.pone.0273376)
Supplement: S7 Table — CoM: Center of Mass. Direct access to these cluster maps is available via our Open Science Framework repository (see Main Manuscript: Source Code and Data Availability). (DOCX) [file pone.0273376.s018.docx]

**S7 Table. Clusters of age and sex related interactions with the performance monitoring model fixed effects separated by affect property**.

|  | **# of** | **Coordinates (CoM)** | | | **Peak** |
| --- | --- | --- | --- | --- | --- |
| **Fixed-effect** | **voxels** | **x** | **y** | **z** | **F-stat** |
| **Valence** |  |  |  |  |  |
| **Affect x Sex** | 169 | -1.2 | 16.8 | 44.7 | 40.5 |
| **nEVC x Age** | 26 | 3.0 | 15.6 | 29.7 | 61.2 |
| **nEVC x Sex x Age** | 98 | 0.5 | 12.7 | 43.3 | 52.2 |
| **PRO x Age** | 305 | 2.5 | 16.7 | 40.7 | 95.1 |
| **PRO x Sex x Age** | 77 | -4.1 | 25.0 | 37.1 | 55.8 |
| **PRO x Sex x Age** | 23 | 9.2 | 20.6 | 43.0 | 54.7 |
| **PRO x Sex x Age** | 18 | -4.9 | 24.0 | 55.9 | 34.0 |
| **Arousal** |  |  |  |  |  |
| **Affect x Age** | 546 | .6 | 19.9 | 42.6 | 83.1 |
| **Affect x Age x Sex** | 22 | 8.7 | 23.2 | 42.5 | 25.7 |
| **Error x Age** | 21 | 8.2 | 22.1 | 42.9 | 30.5 |
| **Error x Age x Sex** | 35 | 8.2 | 23.3 | 41.7 | 22.1 |
| **PRO x Age** | 395 | -0.6 | 17.6 | 43.9 | 88.4 |
| **PRO x Sex** | 47 | -6.7 | 20.1 | 46.3 | 26.9 |
| **PRO x Sex** | 32 | 4.6 | 25.6 | 56.1 | 46.4 |
| **PRO x Sex x Age** | 166 | -3.7 | 21.4 | 42 | 56.2 |
| **PRO x Sex x Age** | 33 | 4.6 | 26.8 | 55.7 | 85.3 |

CoM: Center of Mass. Direct access to these cluster maps is available via our Open Science Framework repository (see Main Manuscript: Source Code and Data Availability).
