# Supplementary material for: Action-value processing underlies the role of the dorsal anterior cingulate cortex in performance monitoring during self-regulation of affect
Source: PLoS One. 2022 Aug 30;17(8):e0273376. doi: 10.1371/journal.pone.0273376 (PMC9426889; doi:10.1371/journal.pone.0273376)
Supplement: S1 File — (DOCX) [file pone.0273376.s019.docx]

**S1 Proof: Direct proof demonstrating that the expected value of control (EVC) is equivalent to a Q-function with a compound reward that incorporates the cost of action.**

| $EVC(s_{t},a_{t})$ | = | $-C\left( a_{t} \right)+ \sum_{i} p\left( s_{i} \right\vert s_{t},a_{t})(r\left( s_{t+1} \right)+\gamma\cdot{max}_{j}EVC(s_{i},a_{j})), from Equations 1 \& 2$[1] |
| --- | --- | --- |
|  | = | $-C\left( a_{t} \right)+ \sum_{i} p\left( s_{i} \right\vert s_{t},a_{t})\cdot r\left( s_{t+1} \right)+\sum_{i} p\left( s_{i} \right\vert s_{t},a)\gamma\cdot{max}_{j}EVC(s_{i},a_{j})$ |
|  | = | $-C\left( a_{t} \right)\mathbb{+ E(}r\left( s_{t+1} \right)\mathbb{)+ E(}\gamma\cdot{max}_{j}EVC\left( s_{t+1},a_{j}) \right)$ |
|  | = | $-C\left( a_{t} \right)\cdot p\left( 1 \right)\mathbb{+ E(}r\left( s_{t+1} \right)\mathbb{)+ E(}\gamma\cdot{max}_{j}EVC(s_{t+1},a_{j}))$ |
|  | = | $\mathbb{E(-}C\left( a_{t} \right)\mathbb{)+ E(}r\left( s_{t+1} \right)\mathbb{)+ E(}\gamma\cdot{max}_{j}EVC(s_{t+1},a_{j}))$ |
|  | = | $\mathbb{E(-}C\left( a_{t} \right)+ r\left( s_{t+1} \right)+ \gamma\cdot{max}_{j}EVC(s_{t+1},a_{j}))$ |
|  | = | $\mathbb{E}\left( {r'}_{t+1}+ \gamma\cdot{max}_{j}EVC\left( s_{t+1},a_{j} \right) \right), where {r'}_{t+1}= \beta_{1}\cdot r\left( s_{t+1} \right)-\beta_{2}\cdot C\left( a_{t} \right)$ |
|  | ≡ | $Q\left( s_{t},a_{t} \right) by definition ADDIN ZOTERO\_ITEM CSL\_CITATION \{"citationID":"16nFS3oL","properties":\{"formattedCitation":"[2]","plainCitation":"[2]","noteIndex":0\},"citationItems":[\{"id":474,"uris":["http://zotero.org/users/3070086/items/WVCCPSSZ"],"uri":["http://zotero.org/users/3070086/items/WVCCPSSZ"],"itemData":\{"id":474,"type":"book","event-place":"Cambridge, MA","publisher":"MIT Press","publisher-place":"Cambridge, MA","title":"Reinforcement Learning: An Introduction","author":[\{"family":"Richard S. Sutton","given":""\},\{"family":"Andrew G. Barto","given":""\}],"issued":\{"date-parts":[["1998"]]\}\}\}],"schema":"https://github.com/citation-style-language/schema/raw/master/csl-citation.json"\}$[2] |

1. Shenhav A, Botvinick MM, Cohen JD. The Expected Value of Control: An Integrative Theory of Anterior Cingulate Cortex Function. Neuron. 2013 Jul;79(2):217–40.

2. Richard S. Sutton, Andrew G. Barto. Reinforcement Learning: An Introduction. Cambridge, MA: MIT Press; 1998.
